# Potential Utility of A Proliferation-Inducing Ligand (APRIL) in Colorectal Cancer

**DOI:** 10.3390/ijms252312496

**Published:** 2024-11-21

**Authors:** Monika Zajkowska, Karolina Orywal, Mariusz Gryko

**Affiliations:** 1 Department of Neurodegeneration Diagnostics, Medical University of Białystok, 15-269 Białystok, Poland; monika.zajkowska@umb.edu.pl; 2 Department of Biochemical Diagnostics, Medical University of Bialystok Clinical Hospital, 15-269 Białystok, Poland; karolina.orywal@umb.edu.pl; 3 Department of Biochemical Diagnostics, Medical University of Białystok, 15-269 Białystok, Poland; 4 Department of Surgical Nursing, Medical University of Białystok, 15-274 Białystok, Poland; 5 1st Clinical Department of General and Endocrine Surgery, Medical University of Bialystok Clinical Hospital, 15-276 Białystok, Poland

**Keywords:** TNFSF13, APRIL, CRC, diagnostics, prognosis, screening, therapy

## Abstract

APRIL (A proliferation-inducing ligand) is a member of the tumor necrosis factor superfamily that is overexpressed in a variety of malignant tumors, including colorectal cancer (CRC). Its key physiological roles include inducing the immunoglobulin switch and ensuring plasmocyte survival. In terms of pathological roles, APRIL antagonism has been identified as a key target in autoimmune diseases and immunoglobulin disorders. As previously demonstrated, several inflammatory processes occur at the site of neoplastic initial stages, and their local symptoms are difficult to detect, particularly in the early stages. That is why we chose to study the current literature on APRIL’s role in the development of colorectal cancer. The main objective of our research was to investigate the role of APRIL in cancer initiation and its usefulness in the detection and therapy of CRC. Interestingly, the findings conducted so far show that the selected protein has a significant potential as a CRC biomarker and treatment target. Importantly, based on its concentration, it is possible to identify CRC patients, but whether the lesion has a benign or malignant nature, indicating the possibility of rapid detection of an ongoing disease process.

## 1. Introduction

The APRIL (A Proliferation-Inducing Ligand) protein is a cytokine that regulates the immune response as well as cell proliferation and survival. In colorectal cancer, APRIL is gaining interest as a possible biomarker and therapeutic target [1]. According to studies, in pathological circumstances such as cancer, the level of APRIL expression may be altered, affecting disease genesis and progression. Understanding the mechanisms of APRIL action in colorectal cancer may lead to new diagnostic and therapy options, as well as offer understanding on the intricate connections between the immune system and colorectal malignancies [2,3,4,5]. Therefore, the aim of our work was to summarize the available literature on APRIL and its involvement in colorectal cancer carcinogenesis, as well as its utility as a possible biomarker and therapeutic target in colorectal cancer.

## 2. Tumor Necrosis Factor Ligand Superfamily Member 13—APRIL

The TNFSF13 gene encodes the APRIL protein (A proliferation-inducing ligand, also known as tumor necrosis factor ligand superfamily member 13 or TNFSF13), which was initially cloned in 1998 [2,6]. This protein is recognized by two cell surface receptors: TACI (transmembrane activator and CAML interactor, also known as tumor necrosis factor receptor superfamily member 13B—TNFRSF13B) and BCMA (B-cell maturation antigen, also known as tumor necrosis factor receptor superfamily member 17—TNFRSF17). TACI recognizes a variety of ligands, including BAFF (B-cell activating factor) and CAML (Calcium-modulating ligand), while BCMA (except for APRIL) also binds BAFF [7]. APRIL has the highest affinity for BCMA receptors and moderate affinity for TACI, whereas BAFF interacts in exactly the opposite way [7]. TACI and BCMA receptors are mostly present on the surface of B lymphocytes and plasmacytes; however, they can also be detached from the cell membrane to serve as decoy receptors that bind BAFF and APRIL. These interactions prompted the discovery of the BAFF/APRIL system 25 years ago, which plays a significant role in the development of immune-mediated disorders such as SLE (systemic lupus erythematosus) and MM (multiple myeloma) [6,7,8].

**Figure 1:**
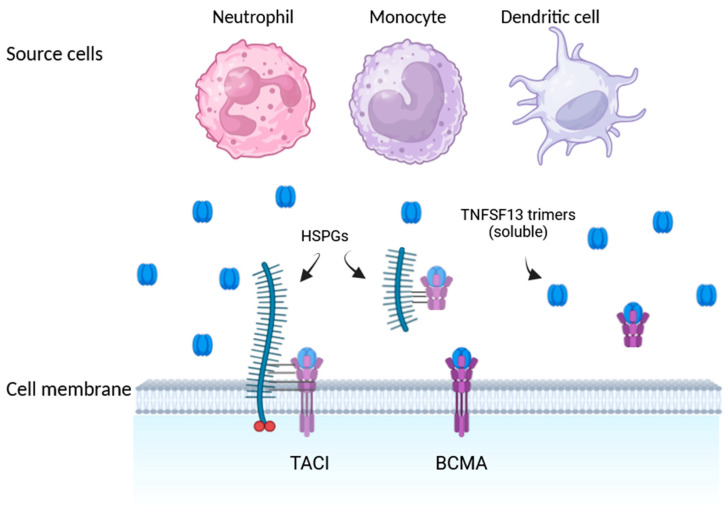
TNFSF13 (APRIL), its sources, and receptors [10]. Description: Neutrophils, monocytes, and DCs are major APRIL producers. Once secreted, APRIL binds to TACI and BCMA. HSPGs have the ability to boost and aggregate APRIL on the target cell surface. Abbreviations: TNFSF13—APRIL protein; HSPGs—heparin sulfate proteoglycans; BCMA and TACI—APRIL-binding receptors.

APRIL (as well as BAFF) belongs to the TNF superfamily, and it is detectable at low levels in healthy tissues but elevated in numerous malignant cell lines. This transmembrane protein has a homotrimeric type II form and is proteolytically processed at a furin protease site, resulting in a soluble form that can be classified and identified as homo- and heterotrimeric [9]. Its primary physiological functions are to induce the immunoglobulin switch and to ensure plasmocyte survival [3]. Under physiological conditions, bone marrow myeloblasts, promyelocytes, myeloblasts, metamyelocytes, band neutrophils, and eosinophils, as well as several leukocytes in tissues and in the periphery, including neutrophils, eosinophils, dendritic cells, monocytes, and macrophages, exhibit the capacity to synthesize and release APRIL [4]. This protein is processed in the Golgi apparatus before being released and is generally found only in a soluble form once it has left the cell’s interior. One exception to this is APRIL-δ, described as a malignant variant of APRIL that remains membrane-bound on leukemia cell progenitors and lacks a furin cleavage site [10]. APRIL can bind to heparin sulfate proteoglycans (HSPGs), which are physically unrelated to TNF receptors, but may boost APRIL signaling at specific locations and aggregate APRIL on the cell surface [11]. Furthermore, TACI can interact in a complex both with HSPGs and APRIL [12]. Figure 1 provides comprehensive details on APRIL (TNFSF13).

Looking into the physiological effects of APRIL, it is clear that the evidence collected so far by utilizing animal models primarily involves pro-inflammatory functions via interaction with the TACI receptor (IgA production, B- and T-cell stimulation) [13,14,15,16,17] and some anti-inflammatory activities (IL-10 generation by B-cells or suppressive action triggered by anti-CD3, both using the TACI receptor), but those have not yet been confirmed in vivo [18,19]. In terms of pathological roles, APRIL antagonism has been identified as an important target in autoimmune illnesses and immunoglobulin abnormalities [3]. Research conducted so far indicates that APRIL has been shown to be produced by tonsil epithelial cells and keratinocytes. Research on the tonsil mucosa has revealed that the predominant source of APRIL in tissues with no signs of infection were keratinocytes, while in the diseased tissue, the main source were neutrophils. Furthermore, it was shown that APRIL expression in keratinocytes is upregulated by pathogens via Toll-like receptors (-2, -4, -7, and -9). It has been demonstrated by later research that neutrophils can constitutively secrete APRIL in addition to producing it in response to inflammation [4]. Different research has shown that APRIL can also be synthesized by brain astrocytes, notably in MS (multiple sclerosis) lesions, to support pathogenic B lymphocyte survival or to alter the growth in polymeric IgA1 plasma cells in the bone marrow in immunoglobulin A nephropathy. This initiated the need to create anti-APRIL therapies [20,21]. APRIL also plays an important role in understanding the relationships that underlie diseases such as multiple myeloma (MM), as it is one of the most important ligands for the BCMA receptor, for which an antibody has been used in MM therapies [22]. The BAFF-APRIL system is also being recognized as a key regulator of B-cell functionality and associated autoimmune disorders such as systemic lupus erythematosus (SLE) and blood malignancies [6]. The role of APRIL and its dependence on BAFF in hematological and solid malignancies is less widely recognized. However, there are studies available describing the involvement of APRIL in certain cancers. For example, in the work by Pelekanou et al. [23], it was noted that APRIL mRNA expression was reduced in breast cancer cells and that those cells secreted APRIL and BAFF in an autocrine manner. However, malignant luminal and basal breast cancer cells, as well as other cells in the tumor microenvironment, were found to release APRIL [24]. According to Zhi et al. [25], gastric cancer (GC) cells exhibit varying degrees of APRIL expression, whereas comparably healthy gastric mucosal cells display minimal levels of APRIL protein. Moreaux et al. [26] have shown that APRIL mRNA is overexpressed in at least eight malignancies when compared to their corresponding normal cells. Furthermore, APRIL-producing cancer cells were identified in 30%, 13%, and 36% of lesions in bladder, ovarian, and head and neck cancers, respectively. On the other hand, one report stated that in most types of cancers (bladder, breast, cervical, endometrial, gastric, head and neck, liver, and lung, as well as colorectal), APRIL mRNA expression is downregulated [27]. This observation suggests that the APRIL produced by tumor cells is not uniformly expressed but rather localized within specific tumor fragments, indicating that these cells do not constitutively produce APRIL, in contrast to neutrophils. The researchers reached a similar conclusion, positing that the increase in APRIL expression observed in their study was primarily due to the infiltration of APRIL-producing neutrophils rather than autocrine synthesis by the tumor cells themselves [28]. Additional investigations into basal cell carcinoma (BCC) cells have further demonstrated that BCC cells are incapable of synthesizing APRIL independently [29].

## 3. Colorectal Cancer

Colorectal cancer (CRC) typically arises as a result of uncontrolled cell development in a specific area of the large intestinal tract [30]. According to the World Health Organization (WHO), there were almost 20 million newly diagnosed cancer cases and 9.7 million cancer-related deaths in 2022, with colorectal cancer (CRC) accounting for 1.926 million new cases and more than 900,000 deaths worldwide. CRC ranks third among women and men in terms of both incidence and death rates, which has changed in recent years, when CRC was ranked second among men in terms of incidence. However, in the absence of gender differentiation, CRC mortality ranks second. Economically advanced countries have 3–4 times greater incidence rates than developing countries, but there is less fluctuation in death due to the second group’s comparatively greater case fatality rate. What is particularly disturbing, as noted by Bray et al., is that there is an approximately 10-fold difference in colon cancer incidence rates by world region in men and women, with the highest rates in Europe, Australia, New Zealand, and Northern America [31].

The majority of colorectal cancers develop by a multistep process that includes a succession of histological, morphological, and genetic changes that occur over time [32]. The most common pathway for CRC development, starting from normal mucosa to adenomatous polyp to invasive cancer, is the Adenoma–Carcinoma Sequence. Understanding the development of colorectal cancer helps in identifying at-risk individuals, implementing preventive measures, and informing screening strategies that can significantly reduce the incidence and mortality associated with this disease [33]. The standard approach for the detection of benign lesions, specifically adenomas (representing primary prevention) and early-stage cancers (in terms of secondary prevention), is screening. The presence of a growing malignancy in the majority of cases is asymptomatic, as patients diagnosed with colorectal cancer do not experience any symptoms of the developing disease. One of the most prevalent signs is blood in or on the stool, which is accompanied by iron deficiency anemia (fatigue and weakness). Other symptoms depend on the underlying tumor’s location. Thus, early detection, which is presently restricted to screening examinations such as FOBTs (fecal occult blood tests) and colonoscopy (method of choice), is critical [34,35]. As endoscopic waitlists for routine CRC screening grow longer, early identification and treatment may become increasingly difficult [36]. That is why, in certain situations, there is the possibility to assess the concentration of tumor-associated proteins in medical laboratories, such as carcinoembryonic antigen (CEA) and, in some cases, carbohydrate antigen 19-9 (CA 19-9), which are also used in cases of patients with CRC, although their diagnostic sensitivity and specificity are considered poor [37]. That is why, most commonly, they are used to provide information about possible recurrence after surgery when determined at the time of excision of the cancerous lesion [34,37]. As a result, many scientists are focusing on the search for novel biomarkers that could facilitate CRC detection at the earliest possible stage of advancement and substantially impact patients’ survival and quality of life, as laboratory diagnostics are one of the quickest ways of receiving a test result, leading to improved outcomes [38,39]. In this work, we also aim to prove the usefulness of APRIL as a potential biomarker for CRC.

The development of CRC is caused by many factors but can also be sporadic or hereditary. Hereditary CRC disorders account for approximately 5 to 10% of all CRCs, 20 to 25% show familial aggregation, and approximately 70% of cases are sporadic [40,41]. All risk factors can be divided into modifiable and non-modifiable. Although we cannot change non-modifiable factors—such as ethnicity, gender, and age, as well as illnesses such as Inflammatory Bowel Disease and inherited illnesses—or their treatment (e.g., treatment with androgen derivatives), it is critical to minimize the number of modifiable factors, the most significant of which is diet [42]. The ‘Western lifestyle’ characterized by higher consumption of red meat, fats, and total calories, along with an increasing life expectancy and population growth, indicates a notable rise in the burden of colorectal cancer (CRC). It is extremely important to maintain a balance between products with a high inflammatory index and products with a lower index. A diet high in fiber, unsaturated fatty acids, omega acids, B-group vitamins, selenium, zinc, magnesium, fat-soluble vitamins, beta-carotene, folic acid, and caffeine/tea could assist in attaining the appropriate balance [43]. In addition to diet (connected also with obesity and diabetes/glucose intolerance), the main modifiable risk factors for CRC are a sedentary lifestyle, smoking (whether active or passive), alcohol intake, and the lately frequently studied gut microbiome. The microbiota biochemically alters the substances ingested by the host, both exogenously and endogenously. Its dysbiosis during colorectal cancer development causes the depletion and/or enrichment of specific gut bacterial species and their metabolic processes. Therefore, maintaining a healthy microbiome is so important due to many reports indicating the involvement of dysbiosis in the development of CRC, especially in younger individuals [44,45].

Any processes that have a significant impact on the increase in inflammation in the body become potential triggers for the initiation of neoplastic processes. As it has been proven before, both during neoplasia accompanying the development of colorectal cancer and other cancers, numerous inflammatory processes are present at the site of neoplastic initiation, and their local symptoms are difficult to observe, especially at the initial stage. Both an unhealthy diet that increases the inflammatory index and dysbiosis of the gut microbiota promote the development of these processes in the colon [46,47,48]. As APRIL impacts immunological cells, we chose to study the current literature on its role in the development of colorectal cancer. Due to the small number of publications available on this topic, it seems extremely important to summarize and determine the current state of knowledge on the utility of APRIL in cancer initiation and its usefulness in the detection and therapy of CRC, which was the main aim of our work.

## 4. Role of APRIL/TNFSF13 Cytokine in Colorectal Cancer

The role of APRIL in tumor development has become a subject of research in the context of the interactions between tumors and the immune system. This is mainly due to the stimulation of tumor cell growth by APRIL, which can support tumor cell proliferation by stimulating signaling pathways responsible for cell growth and survival [1,5]. Interestingly, APRIL can promote immune responses in the tumor microenvironment that are beneficial for its growth while inhibiting responses that could lead to the elimination of tumor cells (especially through interaction with the TACI and BCMA receptors) [5,49]. Some authors describe that APRIL is also involved in cell survival mechanisms, which may limit the therapeutic effect of chemotherapy or immunotherapy [50,51,52]. There have also been reports of anti-APRIL antibodies being used as a treatment in clinical studies. The authors describe the development of a humanized anti-APRIL antibody that is efficient in reducing tumor cell proliferation both in vitro and in vivo. The humanized antibody has the potential to lower the immunoresponse in clinical environments. As the authors point out, this antibody can also be utilized to treat other APRIL-mediated disorders, including Sjogren syndrome, multiple sclerosis, and systemic lupus erythematosus [53]. To date, research on the role of APRIL in neoplastic progression has primarily concentrated on lesions such as breast, gastric, bladder, ovarian, and head and neck cancers. There are relatively few studies that specifically address colorectal cancer [27,28].

### 4.1. The Role of APRIL in Large Intestine Carcinogenesis

Increasing evidence has shown that the variety of APRIL’s signaling responses is shaped by the combined activation of pathways such as PI3K, NF-κB, and MAP kinases exerting different effects on cancer cells, which have been described in Table 1.

Xu J. et al. first identified the promoter region of the APRIL gene and examined the key transcription factor. Deletion investigation of the APRIL gene revealed that it was responsive to specificity protein 1 (Sp1) and nuclear factor kappa B (NF-kB). Overexpression of these proteins boosted its activity, whereas the use of their inhibitors (mithramycin A, an inhibitor of Sp1; and Bay 11-7082, an inhibitor of NF-kB) decreased the APRIL promoter activity [56]. Different authors investigated whether ectopic production of the APRIL protein impacts tumor growth stimulation. Researchers conducted an observation using an animal model and corroborated their assumptions. Furthermore, knocking down APRIL in initial cultures of colon cancer cells, as well as mouse and human CRC cell lines, reduced tumor clonogenicity and in vivo growth, which is in accordance with other publications. Taken together, these findings indicate that both tumor-derived APRIL and APRIL produced by non-tumor cells promote colorectal carcinogenesis [5].

### 4.2. APRIL as a Potential Biomarker of Colorectal Cancer

In addition, research was carried out on the diagnostic usefulness of determining the soluble form of the APRIL protein (sAPRIL) as a diagnostic biomarker. According to Ding W. et al., sAPRIL demonstrated significant diagnostic utility (AUC = 0.854) at a cut-off point of 5.49 ng/mL, outperforming currently utilized tumor markers (CEA and CA 19-9). Furthermore, it demonstrated a positive connection with the aforementioned indicators, and combining CEA and sAPRIL greatly boosted diagnostic sensitivity. The concentration differences between healthy people and those with diagnosed benign changes were not significant, indicating that the appearance of cancer-transformed cells is the only source of its growth. This is incredibly significant information since such factors might be used to predict the malignancy of the changes observed, e.g., during an endoscopy. However, the work was conducted on a small study group, which is why it is suggested to conduct similar studies on a larger scale [57]. Other researchers studied whether APRIL serum levels can predict survival in colorectal cancer patients. The authors assessed the overall survival using Kaplan–Meier survival analysis and Cox proportional hazards ratios. Their findings demonstrated that APRIL serum levels at the time of surgery were linked to advancement of the disease and prognosis in rectal cancer patients, implying that increasing APRIL levels correlate with disease progression [58]. Similar reports were presented by An S. et al. [1], who revealed that high APRIL tissue expression is connected with a significantly higher rate of metastatic lesions. Similar conclusions originate from the work of other investigators who, utilizing the assessment of APRIL tissue expression, determined that in CRC tumor tissue, this protein is closely associated with TNM stage and depth of tumor invasion and that high APRIL expression predicts poor prognosis [59]. The above reports clearly confirm that the APRIL pathway is overexpressed in colorectal cancer tissues and is connected with unfavorable clinicopathological features and a poor prognosis in CRC patients. The authors also concluded that APRIL could serve as a diagnostic or prognostic biomarker for CRC. Other researchers also investigated whether peptides that specifically bind to soluble recombinant human APRIL could be employed in anti-APRIL therapy. Their assumptions were supported by the application of sAPRIL-BP, which inhibited tumor cell proliferation and cell cycle progression in LoVo cells in a dose-dependent manner. In a mouse colorectal challenge model, sAPRIL-BP decreased tumor xenograft growth in nude mice by suppressing proliferation and inducing apoptosis. Furthermore, in an in vivo metastasis model, sAPRIL-BP reduced liver metastasis of colorectal cancer cells, suggesting that it could be used in the treatment of CRC in individuals with high sAPRIL concentrations [60]. It is important to mention that Arévalo B. et al. recently published a paper in which they describe the first dual magnetic beads-assisted immunoplatform for the simultaneous detection of BAFF and APRIL in patients with, among others, CRC. The study shows that the created immunosensors have higher sensitivity and a substantially shorter test duration than those claimed for ELISA kits, allowing for the simultaneous assessment and distinction of healthy people from those with colorectal cancer. This discovery has the potential to be a breakthrough in quick and simple screening diagnostics, allowing for enhanced effectiveness in detecting neoplastic alterations and, as a result, higher cure percentages [61].

### 4.3. APRIL as a Potential Therapeutic Target in CRC

The first publication on APRIL’s significance in the progression of colon cancer was published in 2008, ten years after its discovery. The authors reported their findings on the detection of APRIL expression in colorectal carcinoma tissues, as well as a comparison of the effects of 5-fluorouracil and cisplatin on APRIL expression in colorectal carcinoma SW480 cells. They demonstrated that the protein expression and mRNA levels of APRIL were significantly higher in colorectal cancer tissues, similar to the cell line applied. Due to the fact that expression of APRIL during the use of 5-fluorouracic significantly increases, anti-APRIL therapy may be a vital supplementary treatment to counteract the impact of APRIL induced by antitumor drugs [62]. Another study on APRIL was released a year later. The authors presented a hypothesis that knocking down APRIL reduces the migration and invasion of human colorectal cancer cells. They studied SW480 cell lines and discovered that knocking down APRIL greatly decreased colon cancer cell adhesion, migration, and invasion in vitro. Interestingly, the authors confirmed that reconstituting APRIL expression significantly recovers CRC cell migration and invasion [4]. In their subsequent work on observing the role of APRIL in CRC biological behavior, the authors observed dramatically repressed proliferation ability of human colorectal cancer SW480 cells that were transfected with an siRNA plasmid vector targeting the APRIL gene. Also, recombinant human APRIL, which was used to stimulate human colorectal cancer HCT-116 cells, increased their metastatic and invasive capabilities [63]. Similar research was conducted in which authors revealed that APRIL depletion utilizing RNA interference in the COLO 205 and SW480 cell lines resulted in growth suppression as well as cell cycle arrest in the G0/G1 phase and apoptosis. As a consequence, they unequivocally confirmed that APRIL plays a significant role in the tumor growth of CRC cells’ invasive or migratory activity and may be a valuable therapeutic target [56]. In another study, the same authors provided research findings on the potential role of the APRIL gene in the onset and progression of CRC. They investigated if APRIL knockdown had an inhibitory effect on the development of the SW480 cell line and caused concomitant expression alterations in miRNAs and mRNAs. Wang F. et al.’s findings revealed that siRNA-APRIL can effectively limit the proliferation of tested cells in vitro and in vivo. Interestingly, other miRNAs might be involved in controlling the phenotype associated with the loss of function in APRIL knockdown, highlighting the potential pathways of miRNA–target regulation of the APRIL gene function in CRC cells. Furthermore, siRNA-APRIL shows significant promise as a new gene therapy strategy for APRIL(+) CRC treatment. This was the first reported attempt to employ anti-APRIL treatment on cell lines [64]. These authors additionally conducted research in search of a more potent technique to treat colon carcinoma utilizing gene therapy; specifically, they created a vector containing four shRNAs against the APRIL gene in SW480 cells. They discovered that several shRNA vectors provided a stronger knockdown impact on APRIL than vectors carrying only one APRIL shRNA. This demonstrates that vectors containing numerous shRNAs can be used as a new therapeutic method for malignancies [65]. Also, Ding W. et al. created a unique negative lipidoid nanoparticle encapsulating small interference RNA (siRNA) for selectively silencing APRIL in the parenchyma of a CRC focus in vivo, with absorption via a lipid raft endocytotic pathway, confirming the assumptions of previous authors [66].

In 2011, an original, similar research study was published with the objective of investigating the effects of lentivirus-mediated RNA interference, which could target APRIL, on the chemosensitivity to 5-fluorouracil of a different CRC cell line: LoVo. The authors showed that siRNA-APRIL transfection reduced APRIL expression. After exposure to 5-fluorouracil, the apoptosis rate of siRNA-APRIL-transfected cells spiked, as did the rate of growth inhibition. The scientists determined that their technique efficiently suppressed APRIL expression in LoVo cells and increased the cells’ chemosensitivity to 5-fluorouracil [67].

To sum up, APRIL’s significance in tumor formation is now being studied in the context of tumor–immune system interactions. However, there are only few studies that would comprehensively describe the exact mechanisms of this protein’s action during the development of CRC. As it has been established in the literature so far, most reports indicate that APRIL expression in CRC increases during the ongoing neoplastic process, and its level depends on the stage of advancement. The higher it is, the higher the protein expression and mRNA level observed in both tissue samples and animal models or cell lines. On the other hand, one report stated that in most types of cancers (bladder, breast, cervical, endometrial, gastric, head and neck, liver, or lung, as well as colorectal), APRIL mRNA expression is downregulated. However, the same study confirms that only in two types of cancer, the expression of the mRNA of the cytokine studied is increased (glioblastoma and papillary renal cancer) [28]. However, as the authors of the paper point out, this discrepancy might be connected with the applied research method using different proportions of stromal immune cell infiltrates and should be interpreted with caution. Similar results were presented by Calu V. et al. [68]. As has been demonstrated, neutralizing APRIL, regardless of the manner of knockdown, inhibits the migration and invasion of human colorectal cancer cells. An important achievement was the discovery of an increase in APRIL during 5-fluorouracil therapy, which could result in a decrease in the effectiveness of the treatment, as well as the development of an effective preventative strategy for this increase. It becomes especially alarming that not only APRIL produced during neoplastic transformation by tumor microenvironment cells may contribute to this development, but also APRIL produced physiologically. As a result, it was critical to identify successful anti-APRIL medication (e.g., sAPRIL-BP) and assess the diagnostic use of APRIL in detecting CRC. As demonstrated in this study, sAPRIL concentrations provide potential for the use of this parameter in rapid, routine CRC screening diagnostics, particularly as, based on its concentration, it is not only possible to distinguish between healthy individuals and CRC patients but also to determine the nature of the lesions present in the large intestine, whether benign or malignant. Although the studies on APRIL in the context of tumors are promising, further studies are needed to fully understand the mechanisms of action of this protein and its role in the pathogenesis of CRC.

## 5. Conclusions

Most studies indicate that APRIL expression increases with CRC advancement. The conducted research also shows that lowering APRIL levels can limit the migration and invasion of CRC cells; thus, discovering an effective anti-APRIL treatment is crucial. Most importantly, evidence suggests that sAPRIL concentrations could be useful for rapid screening, helping to distinguish healthy individuals from those with CRC and assess lesion characteristics.

## Figures and Tables

**Table 1:** Summary of signaling pathways of APRIL in CRC.

Signaling Pathway	Effect on Crc Cells	Reference
NF-κB Pathway	Promotes cell survival and inflammatory responses	[54,55]
PI3K/AKT Pathway	Induces tumorigenesis and cell metastasis	[55]
MAPK/ERK Pathway	Stimulates cell proliferation and migration	[56]
TGF-β1/ERK Pathway	Stimulates cell cycle and inhibits apoptosis	[54]

## Data Availability

No new data were created or analyzed in this study.
